# Green Operation Strategies in Healthcare for Enhanced Quality of Life

**DOI:** 10.3390/healthcare11010037

**Published:** 2022-12-22

**Authors:** Albi Thomas, Suresh Ma, Ateekh Ur Rehman, Yusuf Siraj Usmani

**Affiliations:** 1 Amrita School of Business, Amrita Vishwa Vidyapeetham, Coimbatore 641 112, India; 2 Department of Industrial Engineering, College of Engineering, King Saud University, Riyadh 11421, Saudi Arabia

**Keywords:** green operation strategies, healthcare, hospital, green technology, quality of life

## Abstract

Healthcare services have now become a fundamental requirement for all individuals owing to rising pollution levels and shifting lifestyles brought on by fast modernization. The hospital is a specialized healthcare facility where doctors, nurses, and other medical professionals offer their services. Academics and professionals have emphasized green operation initiatives such as green design, green purchasing, green supply chain, and green manufacturing to increase public awareness of environmental problems affecting company operations associated with healthcare for the quality of life. The purpose of this research is to use total interpretive structural modeling and MICMAC (matrix cross multiplication applied to a classification) analysis to investigate and analyze the elements impacting green operations strategies in healthcare. The data are gathered using a closed-ended questionnaire together with a scheduled interview. The components’ interactions are explored using the total interpretive structural modeling technique, and the MICMAC analysis is used to rank and categorize the green operation strategy variables. The study is a novel effort to address and focus on stakeholders, vision and structure, resources, and capabilities. Green operations strategies have only been the subject of a small number of studies in the past, and those studies were mostly addressed at manufacturing-specific green strategies. Thus, by promoting energy efficiency programs, green building design, alternative sources of energy, low-carbon transportation, local food, waste reduction, and water conservation, the health sector can develop multiple key strategies to become more climate-friendly with significant health, environmental, and social co-benefits for quality of life.

## 1. Introduction

Sustainability is among the key issues that operations management is facing in the twenty-first century [1]. Sustainability is a serious challenge that has received a lot of attention in the operations strategy agenda over the past ten years [2]. Presently, the term “green” is commonly used to refer to sustainability. The terms green or sustainable are used for the purpose of understanding the effects on the environment, society, and the economy [3]. There are numerous ways to define the quality of life; however, measuring and including it in research can be challenging. Fallow-field [4] defined health-related quality of life as “the physical functional, social and emotional well-being of an individual”.

The fundamental responsibility of the health industry is to provide high-quality healthcare. However, the health sector is facing environmental problems affecting company operations associated with health care for quality of life. International organizations such as the “World Health Organization WHO; Healthcare without Harm-HCWH” and national agencies have been established to decrease the impact on the planet. These organizations suggest that the health sector’s initial efforts should include simple changes to hospital architecture, more environmentally friendly waste management, the use of safer chemicals, water and energy saving measures, and the purchasing of environmentally friendly goods. The health sector, according to Beloeil and Albaladejo [5], can develop seven key strategies to become more climate-friendly and to promote significant health, environmental, and social co-benefits. These key strategies include promotion of energy efficiency programs, green building design, alternative sources of energy, low-carbon transportation, local food, waste reduction, and water conservation. The performance of the healthcare industry increases when targeted healthcare units create and put into practice hospital operations strategies that consciously and consistently change structural and infrastructural resource aspects [6]. Finding suitable, appropriate, and efficient ways to improve and implement the health sector model in practice while also enhancing its structures, methodologies, and technologies are essential [7] for transforming healthcare to enhance the quality of life.

The literature on green manufacturing and green healthcare demonstrates that there is a lack of research on green operation methods because most prior research has focused on the green practices of supply chain management. The lack of research on green operation methods, green manufacturing, and green healthcare is evident in the literature because the majority of earlier studies were on the green practices of supply chain management. Hospital green operation initiatives, processes, and practices have not been explored in the existing literature. Certainly, the healthcare industry has received very little consideration when it comes to green operation strategies [8]. The research contribution on green operation strategies is currently minimal [9]. Therefore, research into this industry can offer both academics and practitioners useful insights [10]. Operations strategy has received a lot of attention in the field of operations despite being a relatively new branch of operations management, particularly between 1980 and 1990. However, there is no comprehensive framework for sustainability in operations strategy. Gavronski [1] conducted a survey of the literature on sustainable operations strategies, proposed a framework for such a strategy, and pointed out potential areas for further study in the area [1]. There is limited but growing research in the literature on operations strategy that examines the relationship between operations strategy and healthcare performance.

Despite the success in the field of operations strategy, there is still much to explore about how to properly apply this body of knowledge in the healthcare industry [11]. Green operations strategies have only been the subject of a small number of research in the past, and those studies primarily focused on manufacturing-specific green strategies. The healthcare sector receives very little attention when it comes to green operational strategy [8]. The novel initiative of this study is to bridge the gap by developing a theoretical framework for the variables impacting green operations strategies in healthcare organizations. The study’s goals and methodology are developed using total interpretive structural modelling. The following summarizes the study’s objectives.

- To identify the factors influencing green operations strategies in healthcare organizations.
- To analyze the inter-relationships among the influencing factors of green operations strategies.
- To categorize and rank the enablers, MICMAC analysis is used depending on the factors’ dependence power and driving power.

This paper is structured as follows. The subsequent section covers the literature review, past research, factors identified for the study, and its working definition. It is followed by research methodology including data collection, literature on total interpretive structural modelling, and its steps. Section 4 discusses the results and interpretations followed by the managerial/theoretical implications of this study. Finally, the concluding section includes discussion, limitations, and future research directions.

## 2. Literature Review

The triple bottom line approach to sustainability frames the theoretical position of green operations strategy [8]. The growing need to address environmental issues related to company operations has received substantial attention from academics and practitioners, emphasizing the necessity for the development of different green operations strategies [12]. Green operation practices, as defined by Yu and Ramanathan [13], are practices that contribute to the enhancement of environmental performance in the firm’s operations. Green operations strategy, according to Nunes and Shaw [14], is a conscious approach with a long-term primary goal that aims to adapt to environmental demands on services and systems while delivering socioeconomic value. Beyond the manufacturing sector, the sustainability analysis broadened the focus of the green operations plan, making decision-making more difficult [15]. However, successfully adopting these green strategies is frequently a challenging task [12]. Two basic approaches are used to present the theory of green operations: the first, or strategic approach, encourages the environmental consideration issues within the process of making decisions. The second viewpoint encourages green business strategies based on environmental considerations [8,11,14]. Many authors have provided definitions of green operations based on the application of green initiatives [16]. In accordance with the studies and theories on operational strategy, the components of the approach are operational activities and operational competitive criteria, or competitive capacities [17]. Green operations practices are methods used by organizations to increase their sustainability impact. The broad operational procedures will serve as the foundation for the approaches for service delivery, continuous improvement, management of supply chains, and finally operations [16]. Environmental considerations are closely related to the process of developing strategic operational goals [16].

Green operations aim to increase the sustainability impact of their operations by integrating and coordinating sustainability practices with healthcare service and operational processes. The supply chain partners of the focal firm can benefit greatly from implementing operations methods [10]. Operations decisions follow a predictable pattern as a result of operations strategy, which aids in achieving goals when it is driven by a certain organizational strategy. The four main goals of general operations are cost effectiveness, delivery, quality, and flexibility [18]. Business and operational strategies as well as sustainability are closely related. The strategy for the organization’s operations and business must take sustainability into account [19]. The recent pandemic has altered researchers’ focus on the establishment of healthcare systems [20]. According to a recent study [21], smart cities have a greater COVID 19 readiness level and decrease in fatality rate due to the pandemic; however, they fail to perform in aspects of healthcare system sustainability and resilience. Sustainability in operations management refers to the pursuit of considerable environmental and social benefits at a reasonable cost [19]. There is a concept known as “green operations” in relation to environmental management practices [22]. The adoption of green practices serves as a stimulus for green environmental regulations and escalating demands, in addition to being a social obligation [22].

Migdadi and Omari [8] recognized the best practices for green operations strategies in hospitals. Migdadi [23] identified the best practices of green operations strategies around the globe of airlines by interviewing airlines from various locations. The efficient taxonomies of airline green operations plans were investigated by Migdadi [17]. Green operations initiatives in the automotive industry as reflected in the environmental reports of a few corporations were highlighted by Nunes and Bennett [16]. In 2016, Nunes et al. investigated a luxury car manufacturer’s environmental strategy decisions. A main mobile phone manufacturer’s best practices for green operations strategy from 2008 to 2011 were highlighted by Migdadi [9]. According to data from the automobile industry, Liu et al. [10] addressed the special role that supply chain flexibility can play in the effective adoption of green operations strategies. According to Bergmiller and McCright [24], effective management systems are associated with implementation, which is correlated with business outcomes for both Lean and Green programs [24]. The results of the study showed that green and lean initiatives improved company results. The particular importance of supply chain capabilities in the execution of green operations strategies was examined by Liu et al. [12]. The research on sustainability and technology management had a significant gap when it came to methods for sustainable operations, which Nunes et al. [2] addressed. Internal green management and green process design are two key factors for green operations practices, which Yu and Ramanathan [13] explored. They also looked at the relationships between the adoption of green operations practices and its antecedent factors, including stakeholder pressures and subsequent performance outcomes and environmental performance. Nunes and Bennett [25] proposed a paradigm for green operations to assist businesses in the automotive industry in making better environmental decisions. Gupta [18] introduced the idea of environmental management and discussed how it affected the management of operations and output. In their analysis of recent research, Baines et al. [3] provide a set of findings that reflect the state-of-the-art in the field of green production. Saha et al. [26] looked at the reference pricing effect while examining the effects of dynamic retailer investments in green operations. In accordance with these five factors, Gavronski’s [1] study provided a framework for sustainable operations strategy and provided potential for future research. In order to encourage strategic innovation in business, process management, and promote the strategic management of operations for the exploitation of opportunities beyond the buyer–supplier interface, Fiorentino [27] proposed a new conceptual framework based on the firm border perspective. Agrawal and Ülkü [28] investigated if the capacity to upgrade modules led to more profits and less of an adverse environmental impact. The strategy devised by Nunes and Bennett [19] was founded on the idea that environmental advantages and the financial commitments and operating costs necessary for commercial operations are correlated. Functional programs, expanded hospital service capacity, and marketing strategies based on therapeutic techniques were among the green concepts discussed by Setyowati et al. [29]. Usman and Elzabitah [22] examined the empirical green operations practices in order to establish suitable solutions for hospital green operations. They specifically focused on the management of medical waste in the context of the concept of green hospital operations. The impact of a green operations strategy on the productivity of Kenyan tea processing firms was established by Syanda and Getuno [15]. Jha et al. [30] examined research papers on hospital operations management that were published in reputed operations management and service management journals in the year 2000. During COVID-19, Zdemir et al. [31] examined the healthcare system of the United States to assess how well the operations strategy matrix has been implemented.

## 3. Relevant Factors Identified in Literature

### 3.1. F1: Green Operational Actions

Over the past few years, there has been a noticeable rise in concern regarding green operations techniques. Measures have been taken in the healthcare industry with the goals of reducing costs (by employing more environmentally friendly technologies), delaying or avoiding regulatory actions, and bolstering a positive reputation. The operational actions are operational processes. These activities fall under the categories of intra- and inter-organizational. Intra-organizational practices are those pertaining to internal organizational procedures, such as environmental policies, process optimization to lower CO_2_ emissions, or sold garbage. Environmental design, green distribution, and green purchasing are examples of external practices [9]. Implementing green operational actions internally and externally in the healthcare system improves the quality of care offered by the healthcare system while maintaining a reasonable cost; this will increase the possibility of the general public taking advantage of the services and enhancing the quality of life.

### 3.2. F2: Vision and Structure

An organization’s vision outlines what it hopes to become in the future. Visions emphasize the values and objectives at the core of the organization. Structure refers to the framework for delivering care, which includes hospital resources including employees, funding, and equipment. A healthcare system with a strong vision and structure to develop the country’s health offers better usage of resources and healthcare delivery to the public, which includes onsite medical examinations, advice, and disease awareness and prevention measures. This builds the foundation for a healthier population.

### 3.3. F3: Corporate Social Responsibility

Through corporate social responsibility and a self-regulatory business model, a healthcare organization can be socially responsible towards itself, its stakeholders, and the general public. Corporate social responsibility in the healthcare sector is a commitment to promote economic growth, the life of healthcare employees, and the society as a whole. CSR provides a sense of protecting the environment and the society by delivering healthcare services.

### 3.4. F4: Green Technology/IS Support

The process of identifying and attaining an organization’s operational and strategic objectives through the management of its technological capabilities is known as technology management. The identification, selection, acquisition, utilization, training, maintenance, and diffusion of technologies that enable an organization to minimize its effect on the environment are all included in green technology management. Information may be easily and continually accessible, utilized, and shared throughout a supply chain attributable to IT/IS, which can also include some beneficial green operations. It is necessary to successfully integrate IT/IS into current business processes and practices in order to develop this competence, which can be quite challenging. However, a competitive advantage can be anticipated if it is adequately built. An organization’s supply chain process may benefit from IT integration, which could improve performance. It is thought that green operation strategies might be adopted easily with solid IT/IS support [12]. It is obvious that having access to advanced green technologies and measuring tools has made going green a moving objective [18]. Green technology in this tech-era creates numerous advantages to the general public by transmitting the details of a disease and directing patients to engage in proper treatments. The resource usages, managing patients, including patient direct care, and laboratory services are all included in the green technology. The environment is less negatively impacted when technology is used to support attempts to create an optimized process in the development and renovation of healthcare facilities. Additionally, it also improves patient outcomes by shortening waiting hours, using efficient techniques and treatment procedures, and allowing the healthcare professionals to better follow the progress of their patients.

### 3.5. F5: Green Supply Chain Flexibility

The idea of green supply chains introduces sustainability and environmental concerns into the buyer–supplier relationship [25]. Healthcare customers are directly influenced by supply chain flexibility factors. The flexibility of the operations system includes the logistics process, the supplier network, the organizational architecture, and the information system [10]. Flexibility has the advantage of making it easier to execute effective operational strategies that meet healthcare customer expectations and enhance overall performance. Flexibility can be a crucial factor that helps green operations strategies [12]. Integrating environmentally friendly options into supply-chain management research and practice is becoming more and more important [32]. Organizations are seeking to address their major environmental challenges through adopting green practices, which strives to reduce or eliminate waste in all forms along supply chains. This is done in response to competitive, regulatory, and community pressures [33]. The emergence of green supply chain management is seen as a significant breakthrough that aids in the development of win-win strategies by enterprises [33]. Green supply chain implementation will result in a system that is cost-effective, sustainable, and less hazardous to the society. Flexible green supply chain minimizes the energy consumption, resource usage, and waste production by enhancing the quality of health and life [34].

### 3.6. F6: Green Building

Green structures are ones that integrate environmental sustainability with healthcare to create structures with little use of natural resources both during construction and operation, thus achieving resource efficiency. When planning and building an industrial plant, for example, environmental considerations should be included from the very beginning of the operations cycle [25]. Green building enhances the efforts to create optimized workflows in the design of healthcare facilities to reduce waste and the environmental impact. Green building focuses on the long-term well-being, which enhances the healthcare staff and patient outcomes by reducing the overall cost and waiting hours.

### 3.7. F7: Environmental SWOT

An organization’s internal strengths and weaknesses can be correlated with external threats and opportunities using a strength-weakness-opportunities-threat (SWOT) analysis of the environment. Healthcare could benefit more from its investments in environmental policy by using an environmental SWOT analysis. An organization may be able to get better returns from its investments on its environmental strategy by doing an environmental SWOT analysis. Being aware of the green opportunities and practices connected to the main operational processes could encourage the top management to develop a more sustainable business strategy [25]. The environmental SWOT analysis can be used to recognize external threats (such as competitors gaining market share with green services) and opportunities (such as offering an eco-friendly service and resource conservation) and refer them to internal capabilities (such as research and development capabilities, and human resources commitment to protection of the environment) [18]. Environmental SWOT analyzes the recent changes in population health to determine the healthcare service delivery; therefore, this helps to offer better services to the society, enhancing the availability of care to all.

### 3.8. F8: External and Internal Integration

When compared to arms-length relationships, an organization that is able to form collaborative and partnering relationships with its supply chain participants can benefit from a variety of advantages, including increased economic gains through extensive knowledge sharing and decreased transaction costs [12]. Internal integration is the ability of a business to create a connection across all of its operations, including healthcare, so that they may work together to produce outcomes that are satisfactory to both parties. The ability of an organization to collaborate and partner with its stakeholders is referred to as external integration. This type of relationship is best described as an inter-organizational one in which the parties concur to invest resources, work together to achieve shared objectives, exchange knowledge, capabilities, incentives, and accountabilities, and make decisions and address issues together. External and internal integration is necessary for a healthcare organization to create a positive environment for the patients and healthcare workers to provide better outcomes and services. Thus, this helps to enhance the quality of life of the healthcare staff as well as the public.

### 3.9. F9: Stakeholder Pressure

By adapting to stakeholder demands, healthcare organizations may create efficient environmental programs and gain a competitive edge. Demands from stakeholders may prompt the implementation of a range of green operations techniques, from straightforward solutions like internal green management to more complex ones like environmental management. Demands from stakeholders may drive businesses to focus more on environmental issues and incorporate environmental practices into their management strategies. The willingness of a business to employ green operations techniques can be influenced by stakeholder pressures. It is commonly acknowledged that businesses are under pressure to incorporate environmental management practices from a variety of stakeholders, including consumers, suppliers, and competitors [13]. Organizational clients are also very significant stakeholders because their needs can affect how well businesses operate in the area of sustainability strategy. To achieve the performance standards, managers must acquire these competencies to set for the competitive parts of operations [1]. Pressure from stakeholders may have an impact on how receptive an organization is to adopting green operations strategies. Businesses that use a variety of green operations practices in response to stakeholder pressure can encourage strong environmental performance and lessen adverse environmental effects [13]. Stakeholders maintain the healthcare industry’s evolution and update the most recent medical advancements, helping to provide healthcare services based on the best interest of the general population.

### 3.10. F10: Resources and Capabilities

All organizational resources, including capital, labour, and expertise, are finite. One of the crucial aspects of healthcare is allocating the resources and the healthcare capabilities for decision-making in terms of healthcare products and services offered to the public in terms of equality and effectiveness to enhance the quality of health and life. When it comes to operational spending, an organization’s environmental investment selections must adhere to a pattern. It is challenging to create and put into action long-term operations strategy due to a lack of resources. An organization’s operational capabilities, goals, and resource allocation are all governed by its operations strategy. The plan creation and implementation for operating sustainably are constrained by resources, just like all other operational decisions, and are governed by regulations that, while not technically flawless, satisfy many, frequently contradictory objectives. Capital, labour, and expertise are all limited resources inside an organization [1]. Better operational capabilities result from the use of strategies in operational decision-making on issues such as investing in staff development, infrastructure, and structure. The management’s operational capability, which ultimately influences the overall outcome of the organization, is determined by the strategy they apply when allocating organizational resources and organizational activities [30].

## 4. Research Methodology

### 4.1. Data Collection and Interview

The healthcare industry is the primary focus of the current investigation. In this study, we used purposive sampling where the respondents were chosen based on the purpose. The sample size of the study is 30 respondents. Scheduled interviews and closed-ended questionnaires are utilized to collect data for this investigation. A closed-ended questionnaire has been developed to assess the relative importance of all the mentioned elements or pair-wise comparisons. Initially, the questionnaire is revised with the healthcare operations experts and medical professionals. Scheduled interviews are scheduled after the questionnaire has been finalized. Healthcare employees who are currently working in hospitals make up the participants of the final survey. There are 30 members in total for interviews from various states of India. The respondents are selected among a variety of healthcare professionals, including physicians, nurses, physiotherapists, nutritionists, pharmacists, and managers from various departments. One hour is allotted for each interview, of which 50 min are spent to enter the respondent’s data and interview into the required format and 10 min are used to explain the study and the meaning of the factors. Prior to the interview, consent forms are given to the respondents for ethical reasons. The respondent’s privacy and anonymity are safeguarded. For this study, a five-point Likert scale, where 0 denotes no influence and 4 denotes a very high influence, is employed. For instance, if the answer to “Does Factor A Influences Factor B” is “yes,” responders should rate from “1 to 4”; if the answer is “no,” they should rate as “0”.

### 4.2. Relevance of Total Interpretive Structural Modelling

Structural models deal with the modelling process, which places an initial emphasis on identifying the model’s components before analyzing how those elements interact. Due to the interpretive structural model inadequate understanding of direct links, the entire decision-making process could be misrepresented. An interpretive structural-based model can therefore be converted to a total-interpretative-structural-modelling-based model which has been identified as a decision modelling technique [35]. In order to overcome the limitations of conventional interpretive structural-based models, the total interpretative structural model aims to build a strategic theoretical framework by articulating the transitive relations and the justification for the interconnections between the model’s various components. The use of total interpretative structural models to develop a conceptual framework that is contextually relationship based is an enhancement over the traditional interpretive structural model. By using this strategy, specific linkages can be modelled interpretively based on group evaluations of the links between the numerous factors that are involved. The relationships between the elements of a diagraph are better represented using the total interpretative structural methodology [36]. The interpretation of embedded items is handled by the total interpretative structural model through a deliberate, iterative deployment of graph theory. As a result, a directed graph is produced for the intricate system comprising the collection of variables. This helps to transform disorganized mental models into well-organized ones that can support a variety of interpretations. This innovative qualitative modelling technique has been used by researchers in many different academic fields [37].

### 4.3. Steps in Total Interpretative Structural Model

**Figure 1:**
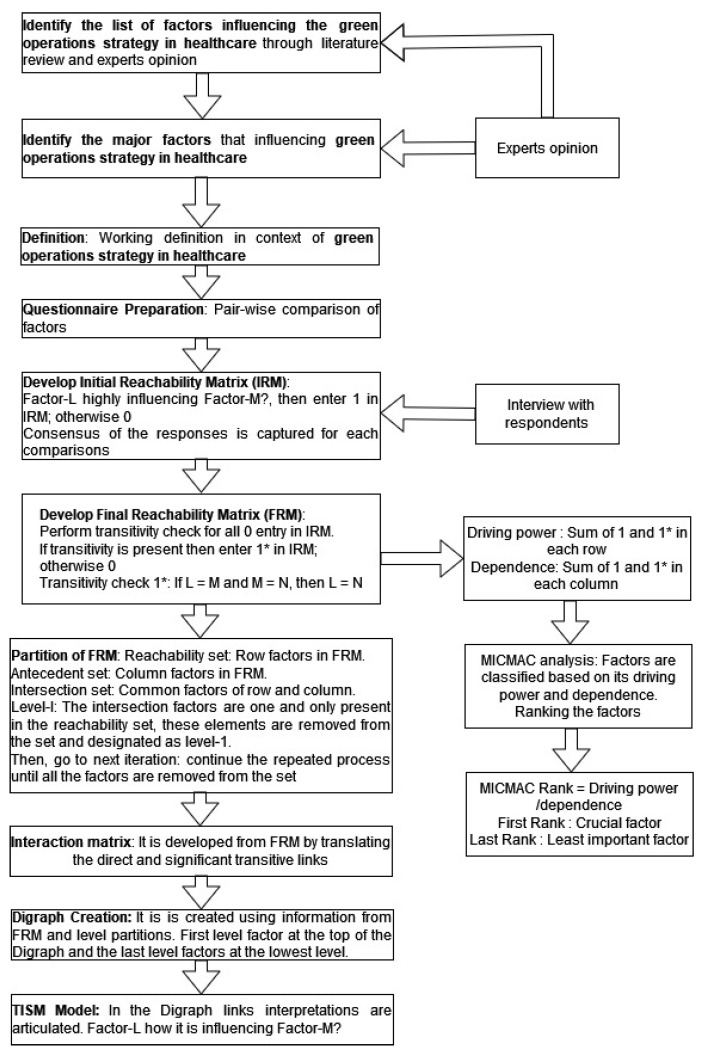
Flow of the total interpretative structural model approach for green operations strategies in healthcare.

To find the key relationships between the variables influencing the green operations strategy in healthcare organizations, a total interpretative structural model is used. In total interpretative structural modelling, an interpretive matrix is used to represent the causal relationships and the expert interpretations of those relationships for in-depth systematic analysis [38]. The total interpretative structural model is an approach used to comprehend how elements interact to influence green operations strategy in the healthcare industry. Many researchers have adopted the total interpretative structural modelling technique for analyzing the interactions between elements in the manufacturing and service industries [39,40]. This paper utilizes the total interpretative structural methodology for the analysis of the interactions between the green operations strategies in healthcare organizations. Figure 1 illustrates the steps in the research methodology [41,42]. The thorough step-by-step analysis is explained in more detail as follows.

The first step is *Identifying and defining the elements*. This phase starts by designing the sample and data collection. In this study, key factors that influence the healthcare organizations’ green operations strategy are defined and identified. These are the factors of the total interpretative structural model and their relationships should be modelled [43]. The stated factors are shown in Table 1.

The second step is to *Develop Initial Reachability Matrix (IRM)*. Determining the contextual relationship between the components that have been found follows next. Here, expert judgment is used to identify pair-wise contextual linkages between each of the elements. As an illustration: if Factor A will have an influence on Factor B, and so forth, along with the appropriate interpretation. Personal interviews and a semi-structured questionnaire with experts are utilized in the framework of this study to analyze the pairwise relationships between components. Thirty healthcare members are selected for interviews. The respondents are selected from among a variety of healthcare professionals, including physicians, nurses, physiotherapists, nutritionists, pharmacists, and managers from various departments. Respondents are selected based on their knowledge and observation capability to enhance the current practices of green operations strategies in hospitals. The consensus of the respondents’ opinions is captured in Table 2.

The third step is to *Construct the Final Reachability Matrix (FRM)*. To create the final reachability matrix, the transitive relations are recognized from the initial reachability matrix. The transitivity rule is used to determine whether the IRM, which is produced from the logical interpretation of the Yes/No connection, contains any potential transitivity [36]. Table 3 contains the final reachability matrix.

The fourth step of the process is *Level partitions in the final reachability matrix*. The elements are level-wise arranged using level partition for the attributes. Three sets are classified viz. reachability, antecedents, and intersection set. All the intersection elements are one and only present in their respective reachability set; these elements are removed from the above three sets and are called level-I elements. After removing the elements from the first level, the reachability, antecedent, and intersection sets are once more determined. The technique is repeated iteratively until all of the elements are removed from the above sets.

The fifth step is *Interaction matrix*. Direct and important transitive relationships assist in creating a matrix. By offering the pertinent interpretation from the knowledge base, it is further developed as an interpretative matrix. It is depicted in Table 4.

Step six includes the *Interpretation of relationship*. The total interpretative structural model has an advantage over the conventional interpretive structural model in this stage since the latter strives to interpret the correlations. The question of interpretation in this study is “How will factor A influence or improve factor B?” It will simplify the process to acquire comprehensive knowledge. The detailed interpretations are captured from the opinions of experts and are depicted in Section 5.1.

**Figure 2:**
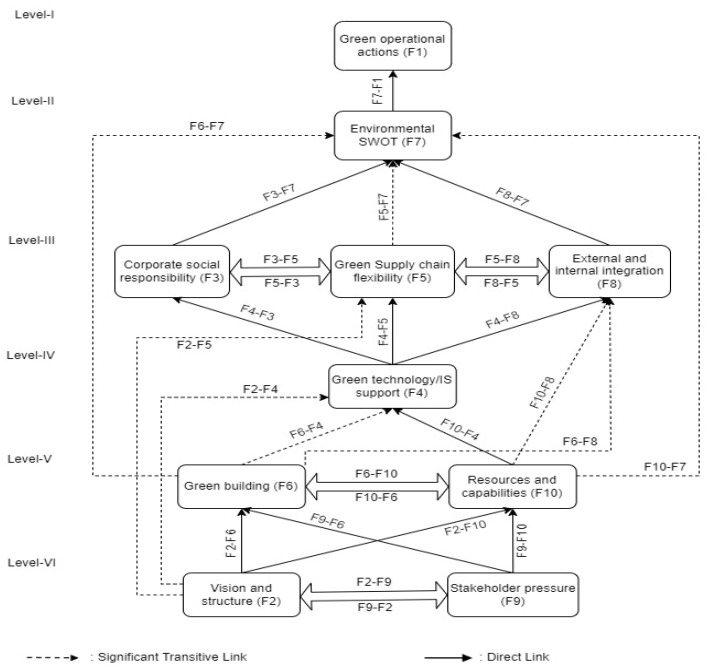
Total interpretative structural model for factors influencing green operations strategies in healthcare.

The final step of the total interpretative structural methodology is the creation of the total interpretative structural model (TISM model), which is the last phase. The links in the obtained digraph display the information from the interaction matrix [35]. The construction of a digraph for elements involves placing each element at its appropriate level and creating directed links according to the relationships shown in the interaction matrix. Only significant transitive links are kept in the digraph. In the digraph, factors at the top of the model are called first level factors and subsequent levels are ranked in ascending order. As a result, the fully justified model is obtained and displayed in Figure 2, while the reasons behind the direct and the significant transitive links are discussed in Section 5.1.

To create the final reachability matrix (Table 3), the transitive relations are recognized from IRM (Table 2). The transitivity rule is used to determine whether the initial reachability matrix, which is produced from the logical interpretation of the Yes/No connection, contains any potential transitivity. From Table 3, direct and important transitive relationships are created as the interaction matrix (Table 4) through the input of experts.

## 5. Results

### 5.1. Interpretation of Total Interpretative Structural Model Di-Graph

The graphical depiction of total interpretative structural analysis of the factors influencing green operations strategies in healthcare is shown in Figure 2.

***Level VI:*** *Level six has two factors, which are factor 2 and factor 9*

*F2 influencing F1:* vision and structure (F2) influencing green operational actions (F1). Although it might be challenging to know where to begin, many organizations are trying to embrace sustainable or green practices. Creating a clear vision and structure for green operational actions is among the most crucial aspects. Organizations can create specific actions once they have a solid understanding of their objectives and constraints. Managers can ensure that the organization is progressing in the correct path by establishing a clear vision and structure. Thus, vision and structure of the healthcare organization helps to enhance the quality of care needed for the population. *F2 influencing F3:* vision and structure (F2) influencing corporate social responsibility (F3). The organization’s vision, which embodies its values and purpose, can inspire employees to participate in corporate social responsibility programs in significant ways. For instance, a business with a sustainability vision may be more inclined to implement corporate social responsibility initiatives that emphasize environmental conservation and public health. *F2 influencing F4:* vision and structure (F2) influencing green technology/IS support (F4). An organization with a clear vision and structure can identify the various technologies and decide which one would work best for its green operations and what is needed for the improvement of population health and healthcare staff well-being. *F2 influencing F5:* vision and structure (F2) influencing green supply chain flexibility (F5). The organization’s vision must be distinct and centered on sustainability. To support the green supply chain, the firm also needs a solid organizational structure. Healthcare organizations can make sure that their green supply chain is adaptable and ready to meet the changing needs of the healthcare market by having a clear vision and a solid framework. *F2 influencing F6:* vision and structure (F2) influencing green building (F6). A clear vision and structure of the healthcare organization can make sure that the green healthcare building satisfies the needs of the environment and also supports the green operational practices. *F2 influencing F7:* vision and structure (F2) influencing environmental SWOT (F7). An effective environmental SWOT analysis requires a clear vision and knowledge of goals. It will help the managers to comprehend how healthcare functions, what effects it has on the environment, and how to improve to maintain green operations. *F2 influencing F8:* vision and structure (F2) influencing external and internal integration (F8). A hospital’s clear vision and structure helps in determining and understanding the healthcare organization’s capacity to integrate all aspects of its operations and also to cooperate and partner with its stakeholders. *F2 influencing F9:* vision and structure (F2) influencing stakeholder pressure (F9). A clear vision and structure aids in developing effective green operational strategies and gaining a competitive edge by adjusting to stakeholder needs. *F2 influencing F10:* vision and structure (F2) influencing resources and capabilities (F10). Due to a lack of resources, developing and implementing a long-term operations strategy is difficult. A healthcare organization’s vision, structure, and operations strategy determine its operational capabilities, objectives, and resource allocation necessary for the society and its well-being.

*F9 influencing F1:* stakeholder pressure (F9) influencing green operational actions (F1). A variety of green operations strategies are implemented in response to stakeholder demands. Organizations are motivated by stakeholder demands to increase their attention to environmental issues and integrate environmental practices into their management plans, which results in green operational actions or activities. Stakeholders help in identifying the recent advances and needs of the society to provide better services to the society to enhance their quality of life through green operational activities. *F9 influencing F2:* stakeholder pressure (F9) influencing vision and structure (F2). The demand from stakeholders can significantly alter the vision and structure of an organization. In reality, decisions about the future of an organization are frequently influenced by this pressure. *F9 influencing F3:* stakeholder pressure (F9) influencing corporate social responsibility (F3). Pressure from stakeholders can encourage hospitals to enhance their green operations and services while also being socially accountable through corporate social responsibility to themselves, their stakeholders, and the general public. *F9 influencing F4:* stakeholder pressure (F9) influencing green technology/IS support (F4). To establish and accomplish the strategic and operational goals of an organization, a variety of green operations techniques and technology management may be implemented in response to stakeholder demands. *F9 influencing F5:* stakeholder pressure (F9) influencing green supply chain flexibility (F5). Healthcare stakeholders encourage the organization to be flexible with its information system, organizational structure, logistics process, and supplier network. *F9 influencing F6:* stakeholder pressure (F9) influencing green building (F6). Demands from stakeholders may drive businesses to focus more on environmental issues and incorporate environmental practices into their management strategies. Stakeholders provide motivation to integrate green building for achieving resource efficiency and green operations. *F9 influencing F7:* stakeholder pressure (F9) influencing environmental SWOT (F7). The demand and pressure from stakeholders would encourage and motivate healthcare management to conduct environment SWOT analysis to identify the opportunities for implementing green operational activities. *F9 influencing F8:* stakeholder pressure (F9) influencing external and internal integration (F8). Stakeholder demand and pressure aids in motivating the organization to work together to implement green operations. Both internal and external integration is required to achieve shared objectives, exchange knowledge, capabilities, incentives, and accountabilities, and make decisions and address issues together. *F9 influencing F10:* stakeholder pressure (F9) influencing resources and capabilities (F10). All organizational resources, including capital, labour, and expertise, are finite. When it comes to operational spending, an organization’s environmental investment selections must adhere to a pattern and it is controlled by the healthcare stakeholders.

***Level V:*** *Level five has two factors, which are factor 6 and factor 10*

*F6 influencing F1:* green building (F6) influencing green operational actions (F1). Environmental design, green distribution, and green purchasing are supported by the green buildings. Green building helps to achieve healthcare goals to reduce costs and employee environmentally friendly technologies, thus fairly providing availability of services to the public. Green structures are ones that integrate environmental sustainability with healthcare to create structures with little use of natural resources both during construction and operation, thus achieving operational practices and efficient healthcare services. *F6 influencing F3:* green building (F6) influencing corporate social responsibility (F3). Green buildings mainly focus on the environmental sustainability and help in creating a corporate social responsibility model for the healthcare organization to improve its operation and quality of service. *F6 influencing F4:* green building (F6) influencing green technology/IS support (F4). For the successful implementation of the technologies required for the healthcare organization, it is necessary to have a sustainable environment friendly structure and design. It is thought that green operation strategies might be adopted easily with solid IT/IS support. *F6 influencing F7:* green building (F6) influencing environmental SWOT (F7). Green building supports the environmentally sustainable operations and the evaluation of its performance to correlate with the external and opportunities using a SWOT analysis. *F6 influencing F8:* green building (F6) influencing external and internal integration (F8). Green buildings create structures with little use of natural resources both during construction and operation, thus achieving resource efficiency to form collaborative and partnering relationships with its supply chain participants that can benefit from a variety of advantages, including increased economic gains through extensive knowledge sharing and decreased transaction costs. *F6 influencing F10:* green building (F6) influencing resources and capabilities (F10). Green structures reduce the use of natural resources to achieve resource efficiency. When planning and building an industrial plant, for example, environmental considerations should be included from the very beginning of the operations cycle.

*F10 influencing F1:* resources and capabilities (F10) influencing green operational actions (F1). Resources and capabilities determine the creation and implementation of plans for sustainable operations. *F10 influencing F3:* resources and capabilities (F10) influencing corporate social responsibility (F3). Corporate social responsibility is designed based on the resources and capabilities an organization has. *F10 influencing F4:* resources and capabilities (F10) influencing green technology/IS support (F4). Based on the availability and allocation of resources, green technology can be implemented. Green technology can be supported only if the healthcare organization has the capability to operate the technology. *F10 influencing F5:* resources and capabilities (F10) influencing green supply chain flexibility (F5). Human resources, technological capability, and financial resources are required for supporting the green supply chain flexibility. If the healthcare organization provides adequate resources, it enhances the supply chain flexibility for green strategy operations. *F10 influencing F6:* resources and capabilities (F10) influencing green building (F6). The healthcare management’s operational capability, which ultimately influences the overall outcome of the organization, is determined by the strategy they apply when allocating organizational resources and organizational activities for green building planning and construction. *F10 influencing F7:* resources and capabilities (F10) influencing environmental SWOT (F7). Better operational capabilities result from the use of strategies in operational decision-making on issues such as investing in staff development, infrastructure, and structure. Thus, resources and capabilities increase the need for environmental SWOT. *F10 influencing F8:* resources and capabilities (F10) influencing external and internal integration (F8). Operational capabilities and enhanced resource management use the operational decision-making in staffing and infrastructure and invests in the internal and external stakeholders.

***Level IV:*** *Level four has one factor, which is factor 4*

*F4 influencing F1:* green technology/IS support (F4) influencing green operational actions (F1). The identification, selection, acquisition, utilization, training, maintenance, and diffusion of technologies that enable an organization to minimize its effect on the environment are all included in green technology management which enhances the green operational actions. *F4 influencing F3:* green technology/IS support (F4) influencing corporate social responsibility (F3). Successful integration of IT/IS into current healthcare processes and practices helps to develop this competence and corporate social responsibility based on the organization’s vision and goals. *F4 influencing F5:* green technology/IS support (F4) influencing green supply chain flexibility (F5). IT support and integration benefit the healthcare organization’s supply chain process which could improve performance through the sharing of information, thus bettering the ability of healthcare professionals to identify and treat patients effectively without long waiting hours. *F4 influencing F7:* green technology/IS support (F4) influencing environmental SWOT (F7). If the information can be easily and continually accessible, utilized, and shared throughout IT/IS, it can also be beneficial for green operations and for conducting SWOT analysis in healthcare to identify the needs of the population. *F4 influencing F8:* green technology/IS support (F4) influencing external and internal integration (F8). Information sharing and defining and achieving the strategic and operational goals of an organization via green technology enhances the relationship between the internal and external stakeholders. It reduces the gap that acts as barrier for external and internal integration.

***Level III:*** *Level three has three factors, which are factor 3, factor 5, and factor 8*

*F3 influencing F1:* corporate social responsibility (F3) influencing green operational actions (F1). Adopting corporate social responsibility programs and models enhances green operational actions through focusing on the environmentally friendly techniques and procedures which can enhance the quality of healthcare services that can uplift the country’s public health. *F3 influencing F5:* corporate social responsibility (F3) influencing green supply chain flexibility (F5). Corporate social responsibility helps in supply chain integration by operating in a way that enhances healthcare quality through stakeholder relationships. *F3 influencing F7:* corporate social responsibility (F3) influencing environmental SWOT (F7). Corporate social responsibility aims to ensure social accountability to healthcare stakeholders which aids in identifying the internal and external strengths, weakness, opportunities, and threats to overcome. *F3 influencing F8:* corporate social responsibility (F3) influencing external and internal integration (F8). Corporate social responsibility enhances the relationship between the external and internal stakeholders by providing awareness regarding green operations.

*F5 influencing F1:* green supply chain flexibility (F5) influencing green operational actions (F1). Flexibility in the supply chain management and activities enhances the green operational actions in healthcare by minimizing waste and efficiently utilizing the resources and capabilities of the organization. *F5 influencing F3:* green supply chain flexibility (F5) influencing corporate social responsibility (F3). To identify and plan the suitable corporate social responsibility practices, healthcare organizations need to integrate environmentally friendly supply chains and to address the greening of operational activities. *F5 influencing F7:* green supply chain flexibility (F5) influencing environmental SWOT (F7). Green supply chain flexibility aids in integrating the environmentally friendly options in healthcare. It also helps to identify the hospital’s internal strengths and weaknesses which can be correlated with external threats and opportunities using a SWOT analysis of the environment. *F5 influencing F8:* green supply chain flexibility (F5) influencing external and internal integration (F8). To integrate supply chains into flexible operations, healthcare organizations are required to integrate the internal and external stakeholders to meet their demands and to provide quality services.

*F8 influencing F1:* external and internal integration (F8) influencing green operational actions (F1). This refers to the ability of a business to create a connection across all its operations, and the ability of an organization to collaborate and partner with its stakeholders. This type of relationship is best described as an inter-organizational one in which the parties concur to invest resources, work together to achieve shared objectives, exchange knowledge, capabilities, incentives, and accountabilities, and make decisions and address issues together, which leads to green operational actions. *F8 influencing F3:* external and internal integration (F8) influencing corporate social responsibility (F3). To identify and understand the suitable corporate social responsibility model for a healthcare organization, it is important for the organization to integrate its external and internal stakeholders and employees. *F8 influencing F5:* external and internal integration (F8) influencing green supply chain flexibility (F5). External and internal integration is required for the supply chain management. It is one of the factors which leads to supply chain flexibility. *F8 influencing F7:* external and internal integration (F8) influencing environmental SWOT (F7). Integration among the internal healthcare employees and external stakeholders is required to analyze the environmental SWOT of a healthcare organization. It enables the healthcare organization to identify weaknesses and transform them into opportunities.

***Level II:*** *Level two has one factor, which is factor 7*

*F7 influencing F1:* environmental SWOT (F7) influencing green operational actions (F1). The environmental SWOT analysis can assist in identifying external risks and opportunities and connecting them to internal organizational strengths and weaknesses. The organization may be able to get better returns from its investments in its environmental strategy by doing an environmental SWOT analysis. On the other hand, being aware of the green opportunities and practices connected to the green operational processes could inspire the top management to develop a green operations strategy.

***Level I:*** *Level one has one factor, which is factor 1*

Green operational actions (F1), which is related to the objective of this study.

### 5.2. MICMAC Analysis

MICMAC involves classifying the factors into four different zones, namely “driving factors”, “autonomous factors”, “dependent factors”, and “linkage factors” [44]. The factors can be explained as below and are depicted in Table 5.

1. *Autonomous factors (Zone-I):* Factors that have weak dependence and weak driving power are known as autonomous factors [45]. In this study, there were no factors falling in the autonomous zone.
2. *Dependent factors (Zone-II):* Factors that have higher dependence on other factors but lesser driving power are known as dependence factors [46]. In this study, environmental SWOT (F7) and green operational actions (F1) are the dependent factors. These factors are influenced when there is a change in other factors.
3. *Linkage factors (Zone-III):* Factors that have a strong dependence and strong driving power are known as linkage factors [47]. These factors establish the connection between the dependence and the driving factors. In this study, green technology/IS support (F4), corporate social responsibility (F3), green supply chain flexibility (F5), and external and internal integration (F8) are the linkage factors.
4. *Driving or Independent factors (Zone-IV):* Factors that have a strong driving power but weak dependence are known as driving factors or independent factors [48]. In this study, vision and structure (F2), stakeholder pressure (F9), green building (F6), and resources and capabilities (F10) are the driving or key factors.

The elements influencing green operations strategies in healthcare are ranked according to the MICMAC analysis [49] in Table 6.

**Figure 3:**
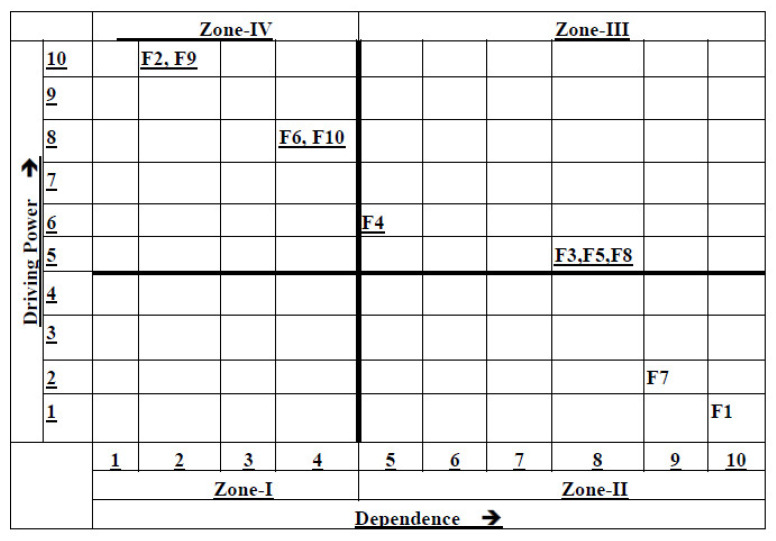
MICMAC graph.

Figure 3 represents the MICMAC graph. Table 6 shows the ranking of the factors influencing green operations strategies in healthcare based on their driving power and dependence power. According to the ranking, vision and structure (F2) and stakeholder pressure (F9) are ranked as 1. Green operational actions (F1) is ranked sixth in the MICMAC analysis ranking. This means that it has a higher dependence on other factors.

## 6. Managerial Implication/Theoretical Implications

### Answering the Questions “What”, “How”, and “Why”

Total interpretative structural models, which are widely used in a variety of domains for conceptualization purposes, address the fundamental concerns of “what,” “how,” and “why” [38].

*“What”:* This study identifies the factors that influence the green operation strategies in healthcare organizations. To answer the “what” question, factors connected to green operations strategies are discovered through a literature review and expert opinions. The identified factors are green operational actions (F1), vision and structure (F2), corporate social responsibility (F3), green technology/IS support (F4), green supply chain flexibility (F5), green building (F6), environmental SWOT (F7), external and internal integration (F8), stakeholder pressure (F9), and resources and capabilities (F10). The revealed aspects include factor relationships that depend on the driving force and provide a response to the inquiry “what.” According to a wealth of research and expert opinions, the “what” question can be resolved by identifying the ten factors that prohibit healthcare organizations from implementing a green operations strategy.

*“HOW and Why”*: This study provides an explanation for “how” the various components interact with one another and “why” such relationships are intended. It is discussed in Section 5 of the study. The MICMAC rank elements address the “how,” whereas the causality implied by link interpretation addresses the “why”. The total interpretative structural model and MICMAC analysis were used to construct tenable interrelationships and hierarchical levels of elements, thus the “how” and “why” questions were addressed. Identifying connections between the ten components provides an answer to the “how” question. The “why” question is addressed by the total interpretative structural model and MICMAC investigations through the interpretations and hierarchical linkages of the elements. This novel study advances our understanding of organizational theory, sustainability, and preparedness for green operations strategies.

Our current study examines how the factors influencing green operations strategies in healthcare organizations interact with one another. This research can help practitioners and management in the healthcare industry to learn how to operate in a more sustainable way and enhance the services provided. The relevance of each aspect may be determined with the help of the total interpretative structural model and MICMAC analysis, which enables organizations to prioritize the most crucial variables first, followed by the other factors. In this study, the first priorities in green operations strategies are vision and structure (F2), stakeholder pressure (F9), green building (F6), and resources and capabilities (F10), making up the driving or key factors. The next priority is given to green technology/IS support (F4), corporate social responsibility (F3), green supply chain flexibility (F5), and external and internal integration (F8), which are the linkage factors. The least priority is given to environmental SWOT (F7) and green operational actions (F1), which are the dependent factors. These factors are influenced when there is change in other factors. As a result, this stream of directions would assist the management in understanding the factors influencing green operations strategies and to prepare for implementing the strategy in healthcare organizations to meet the healthcare demands, to be sustainable, and to provide enhanced quality of care.

## 7. Discussion and Conclusions

It is challenging to define, classify, and analyze the idea of “quality of life”, which is multifaced and complex. It covers many different societal, historical, financial, legal, and environmental dimensions and has a very broad reach. The necessity of healthcare sustainability is becoming more widely recognized. Sustainable development also includes aims that are related to the “quality of life” along with the environment, economic, and social equality [50]. Healthcare services have now become a basic need for all individuals, regardless of age, gender, or culture due to rising pollution levels and shifting lifestyles brought on by fast modernization [33]. The hospital is a specialized healthcare facility where doctors, nurses, and other medical professionals offer their services for enhancing the quality of life [22]. Academics and practitioners have given green operation initiatives, including green design, green purchasing, green supply chain, and green manufacturing, a lot of attention as a way to raise awareness of environmental issues surrounding corporate operations [22]. Reporting best practices for green operations is beneficial because it highlights the outcomes of excellent performance [8]. Businesses of all sizes and from a variety of industries have started to behave more responsibly towards the environment [23]. To increase their competitiveness, several of them have started to develop more environmentally friendly competitive strategies, which have also improved performance [23]. The majority of environmental projects lack clarity; furthermore, several non-environmental issues such as investment or financial viability, consumer happiness, and quality standards also need to be addressed [16]. Sustainability analysis must take into account the nature of the various organizations, processes, and services that make up a society because it is a strategic issue. A difficult principle would entail using a systemic perspective to comprehend how an organization affects the economy, society, and ecological environment [16]. A greater understanding of how better tactics might be developed inside the operations function is required. In order to adapt to environmental demands on service and operation systems while generating socioeconomic value, the green operations strategy is described as “a systematic plan, focused mostly on the long-term” [14]. The formulation of corporate policies and plans at top-tier firms takes environmental considerations into account. Recently, some corporate decision makers have announced their commitment to the environment [18]. According to a study by Gupta [18], the formulation and implementation of green strategies in healthcare organizations should heavily involve the operations manager. In order to comply with new rules, businesses are usually forced to invest in environmentally friendly technologies (such as pollution-control machinery) and methods (such as sampling techniques) [18]. Operations managers face increasing difficulty as sustainable operations strategies develop, which is a key interest of the study. Sustainable business practices have the potential to be key to facilitating a competitive advantage if properly implemented [1]. This study investigated the green operation strategy factors that enhance the quality of life and developed a conceptual model using total interpretative structural modelling and MICMAC analysis. Green operations strategy implementation and execution requires meticulous planning that engages the healthcare organization and society in order to improve the quality of life. Green operation strategies for sustainable development or green transformation will assist in raising the living standards of the society and also address the health requirements for the present and the future. It is important to note why the factors influence one another, and the reasons for this influence are explained in the (Section 5) results. In this study, the first priorities in green operations strategy are vision and structure (F2), stakeholder pressure (F9), green building (F6), and resources and capabilities (F10), which are the driving or key factors. The least priority is given to environmental SWOT (F7) and green operational actions (F1), which are the dependent factors. As a result, this study would assist the management in understanding the factors influencing the green operations strategy and to prepare for implementing the strategy in the healthcare organization to meet the healthcare demand, to be sustainable, and to provide enhanced quality of care. This study model can be applied in the healthcare industry to understand the important factors that influence the green operations strategies for enhanced quality of life.

This study also has a few limitations. According to the respondents, the factor ranking in the total interpretative structural technique changes. The current analysis is primarily concerned with one area, namely the healthcare industry. In order to develop theories, a core structure of enabling links can be built using the total interpretative structural model framework. The post-Covid global circumstances and unstable environment may alter the interrelationships and hierarchical structure of the components. A theoretical model based on expert opinions is developed through a survey. The investigation could be expanded in the future to different areas. Statistical methods such as structural equation modelling and exploratory and confirmatory factor analyses can be used to test this concept. Other approaches, such as performing longitudinal studies to assess green operations strategies and their impact on healthcare performance, should be taken into account for green transformation.

## Figures and Tables

**Table 1:** Identified factors influencing green operations strategies in healthcare.

Sl. No.	Factors	References
1	Green operational actions (F1)	[9]; Experts’ opinion
2	Vision and structure (F2)	Experts’ opinion
3	Corporate social responsibility (F3)	Experts’ opinion
4	Green technology/IS support (F4)	[10,12]
5	Green Supply chain flexibility(F5)	[10,12,25,32,33]
6	Green building (F6)	[25]; Experts’ opinion
7	Environmental SWOT (F7)	[18,25]
8	External and internal integration (F8)	[12]; Experts’ opinion
9	Stakeholder pressure (F9)	[1,13]
10	Resources and capabilities (F10)	[1,30]

**Table 2:** Initial reachability matrix factors influencing green operations strategies in healthcare.

	F1	F2	F3	F4	F5	F6	F7	F8	F9	F10
F1	1	0	0	0	0	0	0	0	0	0
F2	1	1	1	0	0	1	1	1	1	1
F3	1	0	1	0	1	0	1	1	0	0
F4	1	0	1	1	1	0	1	1	0	0
F5	1	0	1	0	1	0	0	1	0	0
F6	1	0	1	0	0	1	0	0	0	1
F7	1	0	0	0	0	0	1	0	0	0
F8	1	0	1	0	1	0	1	1	0	0
F9	1	1	1	1	1	1	1	1	1	1
F10	1	0	1	1	1	1	0	0	0	1

**Table 3:** Final reachability matrix factors influencing green operations strategies in healthcare.

	F1	F2	F3	F4	F5	F6	F7	F8	F9	F10	Driving Power
F1	1	0	0	0	0	0	0	0	0	0	1
F2	1	1	1	1 *	1 *	1	1	1	1	1	10
F3	1	0	1	0	1	0	1	1	0	0	5
F4	1	0	1	1	1	0	1	1	0	0	6
F5	1	0	1	0	1	0	1 *	1	0	0	5
F6	1	0	1	1 *	1 *	1	1 *	1 *	0	1	8
F7	1	0	0	0	0	0	1	0	0	0	2
F8	1	0	1	0	1	0	1	1	0	0	5
F9	1	1	1	1	1	1	1	1	1	1	10
F10	1	0	1	1	1	1	1 *	1 *	0	1	8
Dependence	10	2	8	5	8	4	9	8	2	4	

* Represents transitive links.

**Table 4:** Interaction matrix.

	F1	F2	F3	F4	F5	F6	F7	F8	F9	F10
F1	1	0	0	0	0	0	0	0	0	0
F2	1	1	1	1 *	1 *	1	1	1	1	1
F3	1	0	1	0	1	0	1	1	0	0
F4	1	0	1	1	1	0	1	1	0	0
F5	1	0	1	0	1	0	1 *	1	0	0
F6	1	0	1	1 *	0	1	1 *	1 *	0	1
F7	1	0	0	0	0	0	1	0	0	0
F8	1	0	1	0	1	0	1	1	0	0
F9	1	1	1	1	1	1	1	1	1	1
F10	1	0	1	1	1	1	1 *	1 *	0	1

* Represents significant transitive links.

**Table 5:** Identified factors into MICMAC categories.

Dependence Power			
Driving power		LOW	HIGH
	HIGH	*Class IV: “Driving or independent Factors”* Vision and structure (F2), Stakeholder pressure (F9), Green building (F6), Resources and capabilities (F10)	*Class III: “Linkage Factors”* Green technology/IS support (F4), Corporate social responsibility (F3), Green Supply chain flexibility(F5), External and internal integration (F8)
	LOW	*Class I: “Autonomous Factors”* (No Parameters)	*Class II: “Dependence Factors”* Environmental SWOT (F7), Green operational actions (F1)

**Table 6:** MICMAC rank for factors influencing green operations strategies in healthcare.

Factor	Driving Power	Dependence	Driving Power/Dependence	MICMAC Rank
F1	1	10	0.100	6
F2	10	2	5.000	1
F3	5	8	0.625	4
F4	6	5	1.200	3
F5	5	8	0.625	4
F6	8	4	2.000	2
F7	2	9	0.222	5
F8	5	8	0.625	4
F9	10	2	5.000	1
F10	8	4	2.000	2

## Data Availability

Not applicable.
